# Improving the Glossiness of Cooked Rice, an Important Component of Visual Rice Grain Quality

**DOI:** 10.1186/s12284-019-0348-0

**Published:** 2019-11-27

**Authors:** Seul-Gi Park, Hyun-Su Park, Man-Kee Baek, Jong-Min Jeong, Young-Chan Cho, Gun-Mi Lee, Chang-Min Lee, Jung-Pil Suh, Choon-Song Kim, Suk-Man Kim

**Affiliations:** Crop Breeding Division, National Institute of Crop Science, Rural Development Administration, Wanju, Republic of Korea

**Keywords:** Eating quality, Glossiness of cooked rice, Palatability test, QTL, MAS, Rice

## Abstract

**Background:**

Rice is one of the few cereals consumed as a whole grain, and therefore the appearance of the final milled product, both before and after cooking, strongly influences the consumer’s perception of product quality. Matching consumer preference for rice grain quality is a key component of rice variety development programs, as the quality drives demand, which in turn drives variety adoption, market price, and profitability. The quality of cooked rice is normally evaluated indirectly, through measurement of key elements driving quality as well as more directly by sensory evaluation, but remains a complex trait conditioned by the genetic complexity of factors driving quality, changes wrought by environment, and the complexity of consumer preferences.

**Result:**

In this study, we evaluated 17 traits, including the taste value obtained by glossiness of cooked rice (TV), to explain rice eating quality by statistical methods and identified QTLs associated with TV. To explain the correlation among traits, exploratory factor analysis was performed for 2 years. The overall eating quality (OE) was correlated with TV and protein content loading at the same factor (PA1) in 2017, and there was a relationship between the OE (PA1) and the TV (PA2) in 2018 (PA1:PA2, *r* = 0.3). In QTL analysis using 174 RILs, three QTLs for TV derived from Wandoaengmi6 were detected on chromosomes 4, 6, and 9. The QTL *qTV9* delimited within Id9007180 and 9,851,330 on chromosome 9 was detected in both years, explaining approximately 17% of the variation, on average. Through the use of fine mapping, *qTV9* was delimited to an approximately 34-Kbp segment flanked by the DNA markers CTV9_9 and CTV9_13, and nine ORFs were listed in the target region as candidate genes associated with TV. In the evaluation of *qTV9’s* effect on OE, the lines with *qTV9* showed a significant increase in correlation coefficiency compared to the negative lines. These data will apply to functional analysis on the glossiness and the MAS breeding program to improve the eating quality of *japonica* as a donor line.

**Conclusion:**

In this paper we report a number of QTL associated with changes in glossiness of cooked rice, and these may have utility in the development of MAS in breeding programs with a specific focus on cooked grain quality.

## Background

Rice (*Oryza sativa* L.) is grown in more than one hundred countries worldwide and is the most widely consumed main daily staple for more than 50% of the global population, especially in Asia. The quality of rice used for eating and rice yield potential is one of the main goals of rice breeding programs. Indeed, the market demand for eating quality is continuously increasing around the globe because it is the most important factor in determining the market price (Aluko et al. 2004). However, the genetic complexity of eating quality and the difficulty in accurate evaluation practically constrain how to improve the eating quality in rice breeding programs operated by conventional breeding techniques (Lestari et al. 2009; Rebeira et al. 2014). Moreover, subjective factors, such as eating culture or dietary habits, postharvest practices, and cooking methods, were involved in assessing the taste of rice (Izumi et al. 2007). The facts made it more difficult to develop rice varieties with high eating quality. In this case, molecular technology could be suggested as one solution to address the constraints caused by genetic complexity or selection inaccuracy (Ebadi et al. 2013; Yun et al. 2016).

Based on various factors associated with eating quality, an understanding of consumer preference caused by different backgrounds is also required if improvements are to be made (Champagne et al. 2014; Windham 2014). In the sensory properties of the rice sub-species *indica,* rice aroma is one of most important preferences in determining rice quality (Suwannaporn and Linnemann 2008). Aromatic rice, e.g., Jasmine and Basmati rice, account for 14% of the global rice trade, and these rice types sell at higher prices due to their unique flavor and texture (Childs and Livezey 2006). However, a glossy appearance and a soft and sticky texture are preferred factors when consuming *japonica* rice in temperate Asia (Takeuchi et al. 2007).

Eating quality could normally be assessed by direct and indirect evaluation methods. The factor directly related to rice eating quality is determined by a palatability test using the sensory properties of cooked rice, such as aroma, appearance, sweet taste, and texture (Ramesh et al. 2000; Bett-garber et al. 2001). Therefore, the palatability test by trained panels is the most appropriate evaluation system. However, the results of sensory evaluation are often inconsistent, even among the same samples, and show less selection efficiency when performed at early generation, in which genetic segregation can still occur (Wada et al. 2008; Yun et al. 2016). Then, the physicochemical characteristics, such as alkali spreading value (ASV), amylose content, and protein content, and the glossiness of cooked rice and the rice starch viscosity (RVA) profile have been suggested and used as an indirect method to estimate rice eating quality (Juliano 1971; Bao et al. 2000; Kobayashi et al. 2008; Wada et al. 2008). Starch viscosity and thermodynamic properties are additional properties for evaluating eating quality. Through physicochemical evaluation, studies of eating quality have been performed to identify traits such as amylose content (He et al. 1999; Tan et al. 1999; Bao et al. 2000), protein content (Juliano 1971; Bao 2004), glossiness of cooked rice using a Toyo taste meter (Lestari et al. 2009; Yun et al. 2016) and textural characteristics (Wada et al. 2006; Hsu et al. 2014). The methods have mainly focused on describing eating quality by identifying the correlation among the indirect traits.

Recently, several studies have been carried out to identify the QTLs for eating quality, focusing on these indirect traits because the use of molecular marker technology can contribute to the elucidation of the complexity of quantitative traits or the inheritance of eating quality in rice. Wang et al. (2007) identified 26 QTLs in 2 years to analyze the genetic basis of the cooking and eating quality of rice as reflected by 17 traits. The results revealed that the *Wx* locus also affects the ASV, while the *Alk* locus makes minor contributions to gel consistency and some paste viscosity parameters. Takeuchi et al. (2007) reported that four QTLs on the short arm of chromosome 3 and five QTLs on chromosome 6 were mapped and clustered at the same region of the QTLs for amylose content. To reveal the genetic regions controlling the eating quality of *japonica* rice Koshihikari, QTL analysis was conducted using 92 RILs indicating 43 QTLs on 16 regions across all chromosomes except chromosome 5. The results showed that 37 QTLs from Koshihikari alleles increased the eating quality, 8 QTLs affected the textural characteristics of cooked rice and 3 QTLs affected the amino acid ratio of polished rice (Wada et al. 2008). Using 144 RILs derived from a cross of *indica* combinations, 54 QTLs related to cooking and eating quality were detected for the investigated traits, suggesting that most of the QTLs for the tested traits were clustered close to and controlled by the *Wx* locus and *Alk* on chromosome 6, respectively (Ebadi et al. 2013). The QTLs associated with 12 grain quality traits were identified using 96 introgression lines (IL) derived from a cross between an *O. sativa japonica* cultivar and *O. rufipogon*. Most detected QTLs clustered near *qDTH6* for heading date on chromosome 6, including *qGCR9* for the glossiness of cooked rice on chromosome 9 (Yun et al. 2016). By association mapping using *indica* multi-parent advanced generation intercross (MAGIC) lines, 16 QTLs for ten physicochemical properties were identified related to eating quality in *indica* rice, and nine QTLs on chromosome 6 suggested that GBSSI impacts the overall eating quality (Ponce et al. 2018).

In this study, we evaluated 17 traits related to rice eating quality for 2 years using the RIL with a *japonica* genetic background. Based on the results, correlation analysis was performed to identify the relationship between the glossiness of cooked rice and rice eating quality. In the QTL analysis, the QTLs for the tested traits were identified and anchored on the rice chromosomes. In addition, the target region was further narrowed down by fine mapping using the derived cleaved amplified polymorphic sequence (dCAPS) marker, and the selected QTL introgression lines indicated increasing degrees of OE and TV. The marker developed in this study would be useful for developing *japonica* cultivars with improved eating quality based on glossiness.

## Results

### Evaluation on the TV of Parents and Development of RILs

**Fig. 1:**
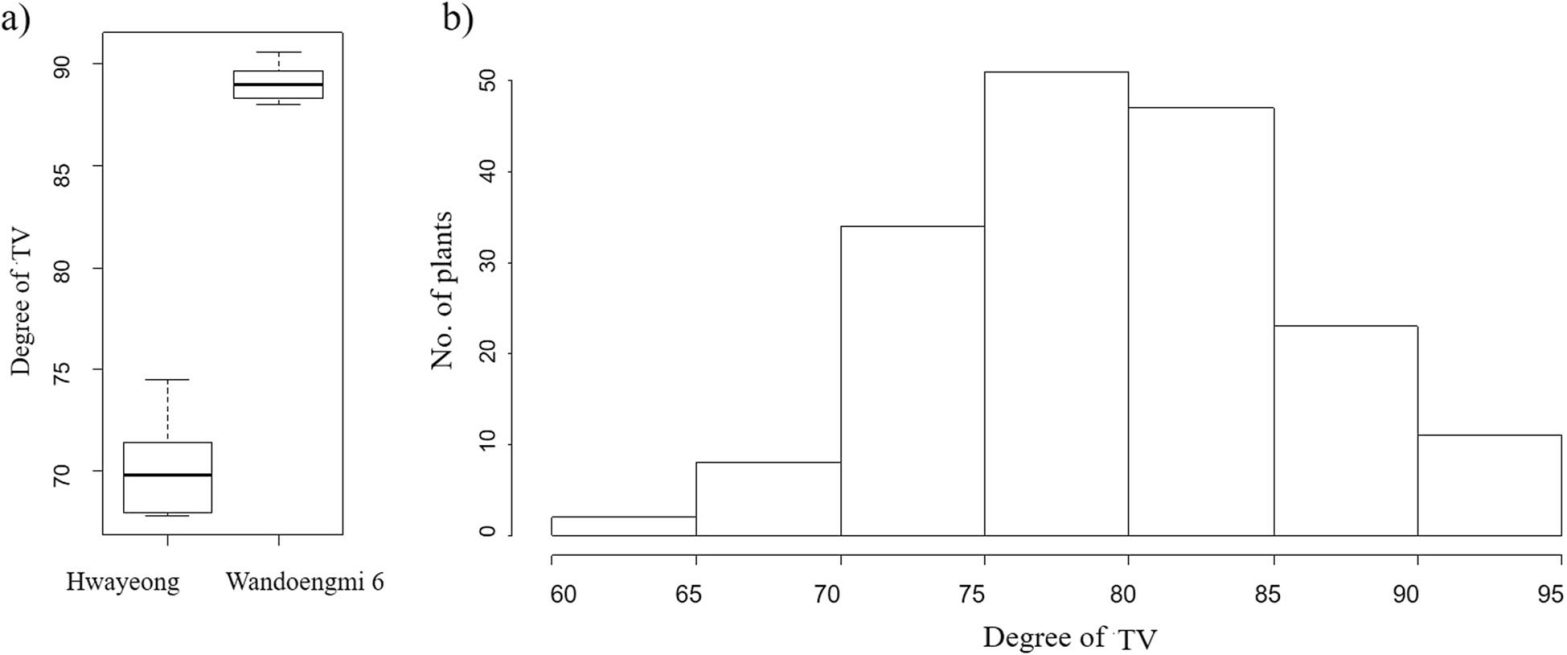
Frequency distribution of 174 RILs derived from Hwayeong and Wandoaengmi6 with parents for the degree of taste value evaluated by a Toyo meter. **a** is a boxplot of parents for the taste value evaluated by Toyo meter (TV), and **b** is a histogram of the population for the taste value

To identify the interaction between TV and eating quality, the TV of the parent Hwayeong and Wandoaengmi6 was evaluated using the TOYO meter (Fig. 1a and Additional file 1: Figure S1). From the result on the TV of Wandoaengmi6, the median was 88.9, and the interquartile range (IQR) was from 87 to 89, while the corresponding values of Hwayeong were relatively low (median = 70, IQR = 67–72). For further study, the 174 RILs were developed by SSD, and the distribution of the TV was analyzed in the RILs (Fig. 1b). The histogram exhibited approximately normal distributions in the population with transgressive segregation.

### Range of Variation for Tested Traits in Parents and the Population

The phenotypic variations of parents and the RILs for 17 traits were evaluated for 2 years (2017–2018) (Table 1). A significant difference was detected between the parents using the *t* test for all tested traits (*P* < 0.05). The parents showed significant differences in all parameters, including grain quality and eating quality, as shown in Table 1, except for AP in the palatability test in 2017. In the case of the cooking quality, pasting temperature (PT), breakdown viscosity (BD), and setback viscosity (SB) were not significantly different both years, especially PV, which was not different between the parents for the years. Year-to-year correlations between the traits in both years were significant (*P* < 0.05 to *P* < 0.001) for all 17 traits, and there was a relatively weak positive correlation in the seven eating-quality traits under the palatability test within the annual periods. From the results of the correlation analysis, the values of TV, PT, CV, and DH showed less variation in the RILs in both years, indicating a moderate positive correlation (*r* > 0.7, *P* < 0.001). The RIL population showed a general normal distribution in the tested traits, with transgressive segregation in both directions for both years.

**Table 1:** Phenotypic variation of 17 traits among parents and the RIL

Traits	Years	Parents			Range of RILs			
		Hwayeong	Wandoaengmi6	T^a^	Mean ± SD	Min.	Max.	C^b^
I. Grain quality (Physicochemical characteristics)								
Protein (%) (Pro)	2017	6.67	6.05	**	7.0 ± 0.7	5.1	9.3	0.64^***^
	2018	6.50	5.87	**	5.9 ± 0.7	4.3	8.4	
Amylose (%) (Amy)	2017	17.5	19.2	*	16.9 ± 1.7	9.5	20.8	0.39^***^
	2018	17.4	19.0	**	18.7 ± 1.1	16.0	21.8	
Glossiness (TV)	2017	68.4	88.5	**	77.1 ± 6.2	56.1	90.4	0.71^***^
	2018	73.0	89.3	**	79.5 ± 5.7	65.2	93.5	
ASV	2017	6.0	7.0	**	6.1 ± 0.8	3.6	7.0	0.58^***^
	2018	5.0	6.0	**	5.5 ± 1.1	2.0	7.0	
II. Eating quality (Palatability test)								
Appearance (AP)	2017	−0.02	0.25	ns	−0.04 ± 0.26	−1.00	0.85	0.26^***^
	2018	0.07	0.57	*	0.02 ± 0.41	−1.11	0.88	
Aroma (AR)	2017	−0.08	0.19	*	−0.01 ± 0.15	−1.00	0.4	0.09^*^
	2018	0.00	−0.07	*	−0.01 ± 0.09	−0.55	0.33	
Taste (EQ)	2017	0.02	0.52	*	0.02 ± 0.33	− 0.85	1.00	032^**^
	2018	−0.14	0.21	*	0.01 ± 0.27	−1.00	0.75	
Stickiness (ST)	2017	−0.30	0.22	*	−0.07 ± 0.32	−1.00	0.71	0.13^*^
	2018	0.00	0.07	*	−0.08 ± 0.38	−1.14	0.75	
Hardness (HD)	2017	−0.16	0.52	*	−0.05 ± 0.41	−1.40	1.00	0.40^***^
	2018	0.07	0.35	**	0.03 ± 0.31	−1.00	1.11	
Overall eating quality (OE)	2017	−0.16	0.55	*	−0.05 ± 0.41	−1.20	1.00	0.41^***^
	2018	0.07	0.28	*	−0.01 ± 0.40	−1.00	1.11	
III. Cooking quality (Starch viscosity)								
Pasting (PT) temperature	2017	73.0	70.4	*	72.1 ± 1.4	68.7	76.0	0.89^***^
	2018	72.7	71.9	ns	73.5 ± 2.1	66.4	78.3	
Peak viscosity (PV)	2017	247.2	247.9	ns	221.5 ± 24.6	137.4	286.1	0.59^***^
	2018	202.9	235.0	ns	219.3 ± 36.3	137.4	280.8	
Hot paste viscosity (HV)	2017	133.9	155.3	*	122.9 ± 22.8	42.8	191.5	0.63^***^
	2018	118.8	151.9	*	129.6 ± 27.6	4.41	188.2	
Cool paste viscosity (CV)	2017	232.2	261.0	*	224.7 ± 29.0	89.5	285.1	0.70^***^
	2018	198.8	242.6	**	212.3 ± 36.1	11.7	269.6	
Breakdown viscosity (BD)	2017	113.3	92.6	*	98.6 ± 15.5	51.8	143.3	0.44^***^
	2018	84.1	83.1	ns	89.8 ± 18.6	15.5	140.9	
Setback viscosity (SB)	2017	−15.0	13.1	*	3.1 ± 19.7	−78.9	54.4	0.57^***^
	2018	−4.1	7.6	ns	−7.01 ± 21.4	−70.0	47.2	
Days to heading (day) (DH)	2017	98	95	*	98 ± 7.9	86.0	121.0	0.94^***^
	2018	101	98	*	98 ± 8.2	84.0	124.0	
Head rice (%) (HR)	2017	95.2	92.9	*	91.9 ± 5.9	63.1	99.2	0.25^*^
	2018	97.0	96.7	ns	79.3 ± 15.5	31.9	98.1	

^a^Difference between the mean value of each parent by *t*-test. ns, *, ** indicates not significant, significant at *P* < 0.05, and significant at *P* < 0.001, respectively

^b^Year-to-year correlation. Significance levels: ^*^P < 0.05, ^**^P < 0.01, and ^***^P < 0.001

### Factor Analysis of Parameters and Correlation of Traits

To explain the relationships among the observed variables of each trait, especially between OE and TA, EFA was conducted for 17 traits evaluated for both years (Table 2). In the scree test for the decision of the number of factors to extract, a total of four factors were taken above the eigenvalues of the factor analysis (data not shown). The results revealed that the contribution of the first three principal axes (PA) was 85% in 2017 and 2018, respectively, and two factors (PA1 and PA2) accounted for over 60% of the variance in the traits related to eating quality. In 2017, all traits in the palatability test and TV and Pro in the physicochemical characteristics were clustered as PA1, and the corresponding loadings of each trait were positive except for the protein. In others, only the loading of the PT and the SB were negatively contributed in EFA for the years. PA1 was correlated with PA4 (*r* = 0.6) and PA3 (*r* = 0.3) in 2017. In 2018, TV was involved in PA2, while Pro was still involved in PA1, and two groups (PA1:PA2, *r* = 0.3 and PA2:PA4, *r* = 0.3) were correlated with each other in 2018 (Additional file 2: Figure S2).

**Table 2:** Factor analysis with orthogonal rotation of the 17 traits evaluated using the RIL population

Traits	2017				Traits	2018			
	PA1	PA2	PA4	PA3		PA1	PA2	PA3	PA4
OE	0.96	0.05	0.22	0.07	OE	0.98	0.16	0.11	0.04
EQ	0.87	0.13	0.23	0.08	HD	0.93	0.16	0.08	0.03
HD	0.84	0.1	0.23	0.11	ST	0.83	0.24	0.12	0.04
ST	0.81	−0.09	−0.09	0.08	EQ	0.83	−0.04	− 0.09	− 0.18
AP	0.66	0.2	0.26	−0.01	AP	0.81	0.04	0.06	0.06
AR	0.51	0.21	0.34	0.03	AR	0.45	0.02	−0.01	−0.07
TV	0.47	−0.17	0.27	0.08	Protein	−0.38	−0.28	0.32	0.19
Pro	−0.65	0.14	−0.39	−0.1	PT	−0.13	− 0.92	0.23	− 0.12
PV	0.12	0.89	−0.1	0.36	DH	0.08	0.86	−0.16	0.19
HV	0.03	0.9	−0.12	−0.24	ASV	0.02	0.7	−0.1	0.07
CV	0	0.98	0.08	−0.2	TV	0.35	0.65	−0.19	0.13
PT	−0.3	0.25	−0.81	0.08	Amylose	0.03	0.64	0	0.09
DH	0.19	−0.12	0.79	−0.11	HR	0.1	0.54	0.28	0.05
ASV	0.16	−0.04	0.68	−0.17	CV	0.08	0.05	0.99	0.06
HR	0.2	0.2	0.49	0.26	HV	0.05	−0.17	0.94	0.04
Amy	0.22	0.24	0.39	0.04	PV	0.08	−0.21	0.86	−0.47
BD	0.13	0.06	0.02	0.98	BD	0.09	−0.15	0.2	−0.93
SB	−0.15	0.37	0.25	−0.74	SB	−0.02	0.42	0.18	0.87
Proportion Explained	0.38	0.25	0.23	0.15	Proportion Explained	0.35	0.28	0.23	0.16
Cumulative Proportion	0.38	0.62	0.85	1	Cumulative proportion	0.34	0.62	0.85	1

*TV* taste value evaluated by Toyo meter, *AP* grain appearance of cooked rice, *AR* grain aroma of cooked rice, *EQ* eating quality of cooked rice, *ST* stickiness of cooked rice, *HD* hardness of cooked rice, *OE* overall eating quality in palatability test, *Pro* protein contents of head rice, *Amy* amylose contents of head rice, *ASV* alkali spreading value, *DH* days to heading in 2017(2018), *HR* percentage of head rice, *PT* peak temperature, *PV* peak viscosity, *HV* hot paste viscosity, *CV* cool paste viscosity, *BD* breakdown (BD = PV - HV), SB: set back (SB = CV - PV)

The correlation value among 13 traits involved in PA1 and PA2 was summarized and generally consistent for 2 years when it was analyzed along with the OE (Additional files 3: Figures S3, S4). From the analysis, some traits (HD, ST, and EQ) of the palatability test showed a strong positive correlation (*r* > 0.8) with OE, while Amy, DH, and HR showed a weak positive correlation (*r* < 3.2) with OE (Table 3). Among the physicochemical characteristics, TV and Pro showed a relatively moderate correlation with OE. In the case of the TV, the mean correlation coefficiency was observed to be the largest for Pro (*r* = − 0.51).

**Table 3:** Correlation coefficients among traits associated with eating quality in 174 RILs for 2017 and 2018

Traits	Years	TV	Pro	EQ	ST	HD	OE	Amy	DH	HR
TV	2017		−0.58^***^	0.39^***^	0.50^***^	0.39^***^	0.48^***^	0.19^*^	0.30^**^	0.23^*^
	2018		−0.44^***^	0.30^**^	0.38^***^	0.37^***^	0.41^***^	0.58^***^	0.63^***^	0.39^***^
Pro	2017	−0.58^***^		−0.60^***^	− 0.62^***^	−0.60^***^	− 0.67^***^	−0.34^***^	− 0.42^***^	−0.23^*^
	2018	−0.44^***^		−0.49^***^	−0.31^**^	− 0.35^***^	−0.35^***^	− 0.22^*^	−0.31^**^	0.06
EQ	2017	0.39^***^	−0.60^***^		0.62^***^	0.88^***^	0.93^***^	0.36^***^	0.30^**^	0.39^***^
	2018	0.30^**^	−0.49^***^		0.62^***^	0.88^***^	0.77^***^	0.01	0.02	−0.08
ST	2017	0.50^***^	−0.62^***^	0.62^***^		0.61^***^	0.73^***^	0.04	0.10	−0.02
	2018	0.38^***^	−0.31^**^	0.62^***^		0.88^***^	0.92^***^	0.26^**^	0.22^*^	0.31^**^
HD	2017	0.39^***^	−0.60^***^	0.88^***^	0.61^***^		0.92^***^	0.37^***^	0.32^***^	0.43^***^
	2018	0.37^***^	−0.35^***^	0.88^***^	0.88^***^		0.95^***^	0.31^**^	0.21^*^	0.23^*^
OE	2017	0.48^***^	−0.67^***^	0.93^***^	0.73^***^	0.92^***^		0.34^***^	0.33^***^	0.36^***^
	2018	0.41^***^	−0.35^***^	0.77^***^	0.92^***^	0.95^***^		0.31^**^	0.21^*^	0.24^*^
Amy	2017	0.19^*^	−0.34^***^	0.36^***^	0.04	0.37^***^	0.34^***^		0.23^*^	0.31^**^
	2018	0.58^***^	−0.22^*^	0.01	0.26^**^	0.31^**^	0.31^**^		0.70^***^	0.64^***^
DH	2017	0.30^**^	−0.42^***^	0.30^**^	0.1	0.32^***^	0.33^***^	0.23^*^		0.41^***^
	2018	0.63^***^	−0.31^**^	0.02	0.22^*^	0.21^*^	0.21^*^	0.70^***^		0.48^***^
HR	2017	0.23^*^	−0.23^*^	0.39^***^	−0.02	0.43^***^	0.36^***^	0.31^**^	0.41^***^	
	2018	0.39^***^	0.06	−0.08	0.31^**^	0.23^*^	0.24^*^	0.64^***^	0.48^***^	

Significance levels: ^*^P < 0.05, ^**^P < 0.01, and ^***^P < 0.001

### Linkage Map and QTL Analysis

Of the 7098 SNPs tested, 1024 showed a polymorphic pattern between *japonica* parents. Before linkage analysis, SNPs stacked in the same linkage position with 0 cM intervals were first removed. A total of 468 SNPs were eventually selected to be anchored on the 12 chromosomes (Additional file 4: Figure S5). The polymorphism rate ranged from 4.33 to 29.23%, with a mean of 14.42%, and on average, approximately 41 SNPs were anchored on each chromosome (Additional file 5: Table S1). The linkage map resulted in a total length of 1064 cM and an average distance of 2.1 cM within the flanking markers. On the linkage map, there were two breaking gaps, which means that the distance of flanking markers was greater than 50 cM apart on chromosomes 4 and 5. The gaps were produced by the absence of markers in the regions because of the similarity of the genetic background caused by *japonica* to *japonica* combination.

**Fig. 2:**
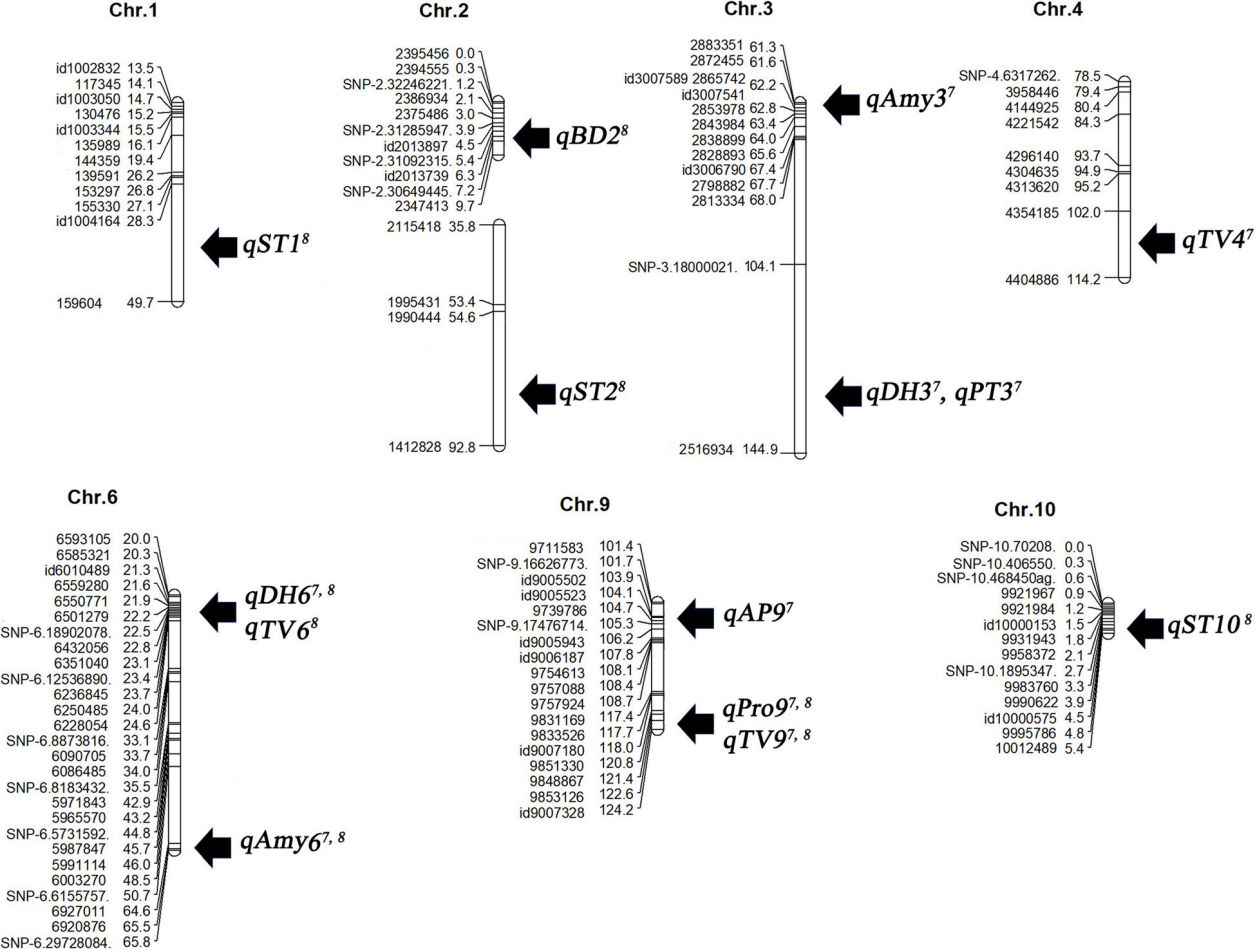
Location of QTLs for eating and cooking quality traits detected in the linkage map on chromosomes 1, 2, 3, 4, 6, 9 and 10. The marker names and linkage distances are shown on the right of each chromosome, and each QTL is presented to the right of the linkage map. Superscripts 7 and 8 indicate the years 2017 and 2018, respectively, in which the QTL is identified

From the QTL analysis based on the linkage map, a total of 14 QTLs associated with traits of eating quality were identified and mapped at an empirical threshold of LOD > 2.40 for the study years (Table 4). Of the tested traits, the QTLs of TV, Pro, DH, Amy, ST, AP, PT and BD were detected on chromosomes 1, 2, 3, 4, 6, 9, and 10 using inclusive composite interval mapping (ICIM) (Fig. 2). Only the four QTLs, *qTV9, qPro9, qDH6*, and *qAmy3* on chromosomes 9, 6, and 3, were continuously detected for the experimental periods. The others (*qAmy3*^*7*^*, qDH3*^*7*^*, qPT3*^*7*^*, qTV4*^*7*^*, qAmy6*^*7*^*,* and *qAT9*^*7*^ in 2017 and *qST1*^*8*^*, qBD2*^*8*^*, qST2*^*8*^*, qTV6*^*8*^*,* and *qST10*^*8*^ in 2018) were detected only in either experimental year of 2017 or 2018 (Fig. 2). For TV, all three QTLs on chromosomes 4, 6, and 9 revealed a positive influence on the alleles derived from Wandoaengmi6. *qTV9*, with an LOD score of 4.96 within 9,848,867 and 9,851,330 and 4.03 within Id9007180 and 9,851,330 was detected on chromosome 9, respectively, explaining 14.36 and 13.95% of phenotypic variation (*R*^*2*^) in ICIM analysis, respectively. In addition, *qTV6* and *qTV4*, with LODs of 2.86 and 2.83, respectively, were detected within flanking markers (6550771–6,501,279 and 4,354,185–4,404,886) on chromosomes 6 and 4 with *R*^*2*^ values of 9.34 and 9.49, respectively.

**Table 4:** QTLs identified for the traits of rice eating and cooking quality for 2 years in the RIL population

Traits	QTLs	Chr.	Position (cM)	L-marker	R-marker	Years	LOD	*R*^*2* a^ (%)	Add ^b^
TV	*qTV9*	9	121	9,848,867	9,851,330	2018	4.96	14.36	2.40
				Id9007180	9,851,330	2017	4.03	13.95	2.01
	*qTV6*	6	22	6,550,771	6,501,279	2018	2.86	9.34	2.11
	*qTV4*	4	114	4,354,185	4,404,886	2017	2.83	9.49	1.70
Pro	*qPro9*	9	121	9,851,330	9,848,867	2018	3.78	15.61	−0.27
						2017	3.61	16.00	−0.19
DH	*qDH6*	6	22	6,550,771	6,501,279	2018	12.29	37.72	5.76
						2017	9.63	27.78	4.41
	*qDH3*	3	135	SNP-318000021	2,516,934	2017	4.77	19.92	3.10
Amy	*qAmy3*	3	62	2,872,455	Id3007589	2018	3.00	5.53	0.65
						2017	3.53	12.70	1.12
	*qAmy6*	6	65	6,927,011	6,920,876	2017	4.41	12.70	1.17
ST	*qST1*	1	29	Id1004164	159,604	2018	3.46	10.42	0.10
	*qST2*	2	92	1,990,444	1,411,125	2018	2.91	8.75	0.08
	*qST10*	10	4	9,990,622	Id0000575	2018	3.94	11.62	−0.10
AP	*qAP9*	9	104	Id9005502	Id9005523	2017	2.62	10.67	−0.06
PT	*qPT3*	3	144	SNP-3.48000021	2,516,934	2017	3.33	5.33	−0.44
BD	*qBD2*	2	7	Id2013739	SNP-2.30649445	2018	2.51	10.42	5.59

^a^Percentage of phenotypic variation explained by the QTL

^b^Additive effect

### Genetic Improvements in Eating Quality by *qTV9*

**Fig. 3:**
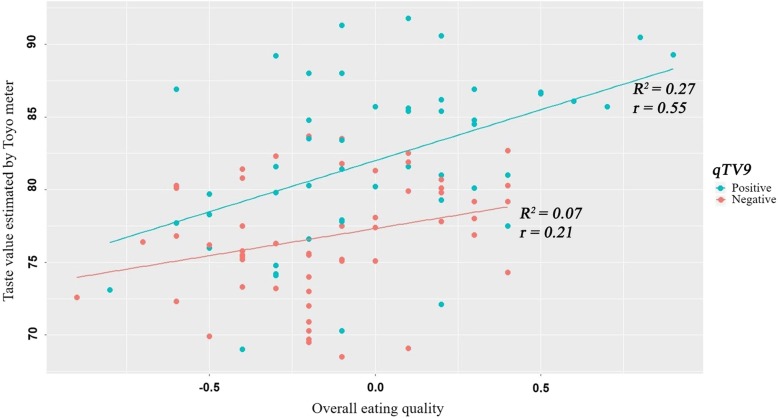
Distribution of the QTL, *qTV9*, on the scatter plot of taste value and overall eating quality (OE) for correlation analysis. The green spots indicate lines with *qTV9,* and the red spots indicate lines without the QTL. The linear lines on the plot show the correlations for significance between the taste value and OE of lines distinguished by the two colors

Before verifying the effectiveness of the QTL, the 110 RILs were selected to minimize environmental errors, considering a range of DH (86 to 106) and less variation in TV for the experimental years. The distribution of OE was analyzed according to the presence of the QTL *qTV9* (Fig. 3). In the case of the positive *qTV9,* the introgression lines with *qTV9* showed a significant correlation (*r* = 0.55, *P* < 0.001) and a determination coefficient (*R*^*2*^ = 0.27, *P* < 0.001) between TV and OE, while the negative introgression lines showed a weak correlation and a determination coefficient (*R*^*2*^ = 0.07, *P* < 0.01) within the traits (*r* = 0.21, *P* < 0.05). The result revealed that the presence of *qTV9* positively increased the TV in the lines, and it was also affected to improve the OE of the introgressed line.

### Delimitation of the Physical Location of *qTV9*

**Fig. 4:**
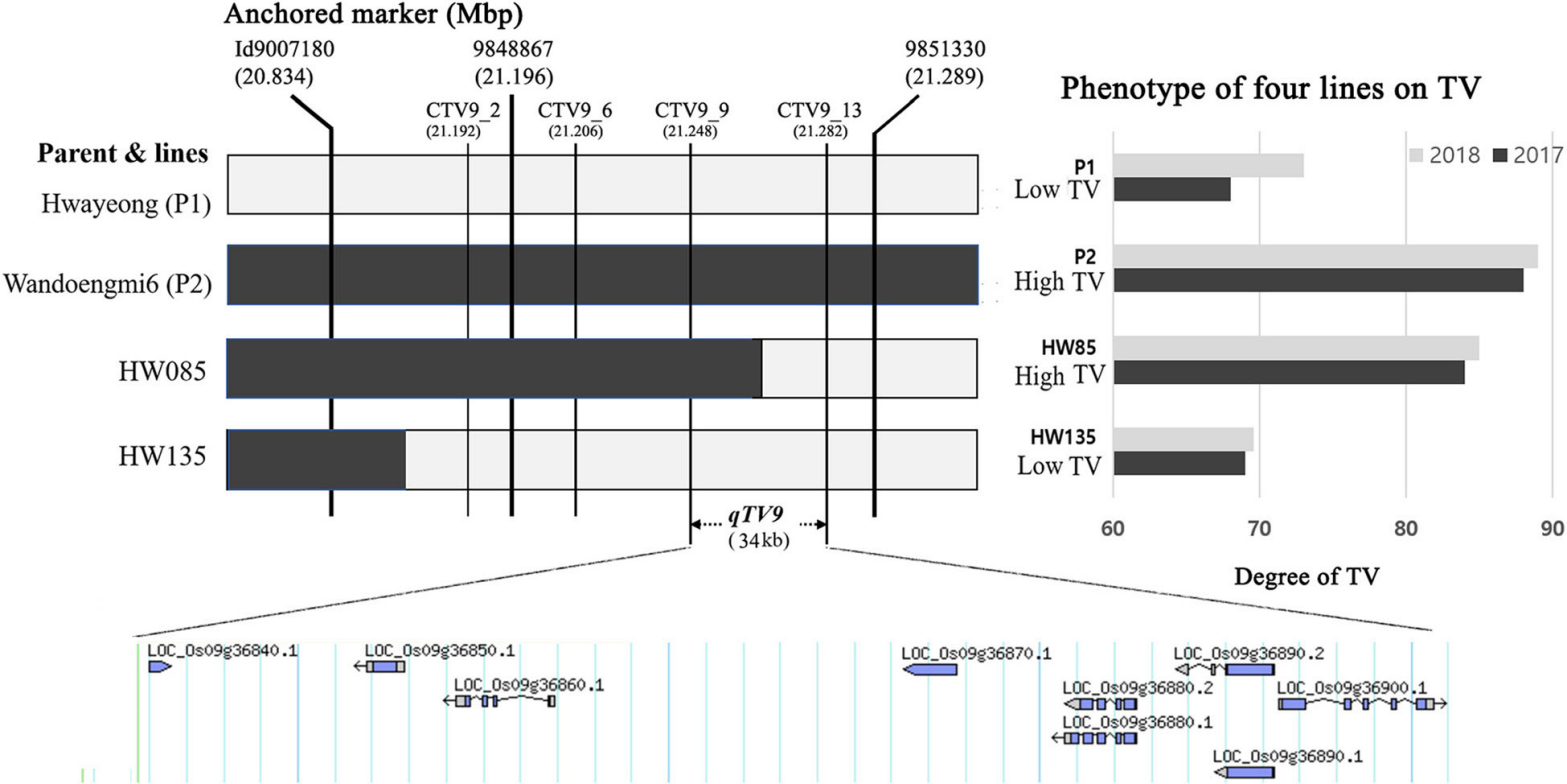
Identification of the target region for the taste value of cooked rice using graphical dissection of the two recombinant lines HW85 and HW135. White and black bars on the graph indicate allele patterns derived from Hwayeong and Wandoaengmi6, respectively. The left graph shows the degrees of the taste value on parents and selected two recombinants. P2 and HW85 showed the same phenotype with relatively high degrees, and P1 and HW135 had low degrees

From the QTL analysis, the QTL *qTV9* was designated by 20.83 and 21.29 Mbp on chromosome 9 in 2017 and by 21.17 to 21.29 Mbp in 2008, respectively (Table 4 and Fig. 4). Among the tested lines, five recombinant lines showing discordance by genotype and phenotype data were observed, and two recombinants (HW085 and HW135) were finally selected to delimit the position because the two were able to be significantly distinguished by their degree of TV. For dissection of the *qTV9* locus, a total of 16 CAPS markers were designed based on the result of the whole-genome resequencing (WGR) using the parents, and five markers showing polymorphism between the parents were selected (Additional file 6: Table S2) to be anchored on the target region, except for CTV9_6. The target region was narrowed to approximately 34-Kbp segments delimited by the flanking markers CTV9_9 and CTV9_13 (Fig. 4). Nine ORFs based on the MSU Osa1 Release 7 located in the target region were identified in the target region as candidate genes associated with the TV of cooked rice (Additional file 7: Table S3).

### Development of the *qTV9* Introgression Lines

To select promising lines for improving eating quality, we started to evaluate agronomic traits and grain quality using RILs at F_6_ generation. Through phenotypic selection, physicochemical characteristics and palatability test, elite lines were continuously selected for each breeding generation. Ten breeding lines were selected at F_8_ generation and confirmed QTLs in the selected lines using QTL validation test. Of them three lines with *qTV9* (HW148, HW184, and HW191) were finally selected as promising lines associated with eating quality (Table 5). For DH, the selected were similar to the parents, and for PL and NP, the degree was not much different within the variation of parents, ranging from 21.1 to 22.8 and 9.6 to 10.6, respectively. CL increased in general on the side of Wandoeognmi6. In Pro, Amy, and ASV, the values of the three lines were distributed at the level of the normal *japonica* cultivar in Korea, even though the amylose contents were biased to Wandoaengmi6. In the case of TV, the values showed a definite increase compared to Hwayeong, according to the presence of *qTV9*. Grain appearance and other traits of the selected three lines were also evaluated (Additional file 8: Table S4). The results reveal that resistance to BL and BB and panicle type also improved with eating quality.

**Table 5:** Comparison of parents and selected promising three lines on agronomic performance and physicochemical characteristics including *qTV9*

Parents & Lines	Major agronomic traits				Physicochemical characteristics					QTLs (*qTV9,qTV6,qTV4)*
	DH (day)	CL (cm)	PL (cm)	NP	TV	OE (−3 − + 3)	Pro (%)	Amy (%)	ASV (1–7)	
Hwayeong	99.5a ^a^	73.4b	21.1b	11.7a	68.4b	−0.16b	6.67a	17.5c	6.0ab	-, −, − ^b^
Wandoaengmi6	96.5a	99.4a	19.8c	9.4b	88.4a	0.55a	6.05a	19.1bc	7.0a	+, +, +
HW148	102a	98.4a	22.8a	10.6ab	83.5a	0.64a	6.54a	21.32ab	6.3ab	+, +, +
HW184	98.5a	94.6a	21.6b	10.0b	87.8a	0.65a	5.99ab	19.98ab	7.0a	+, +, +
HW191	97.5a	92.8a	21.1b	9.6b	86.5a	0.75a	5.38b	21.58a	5.7b	+, +, +

^a^Duncan Multiple Range Test (DMRT) of agronomic traits of tested lines. Means followed by the same letter are not significant at the 5% significant level

^b^“-” is absent of QTL and “+” QTL is present

## Discussion

Rice eating quality is one of the main objectives of the *japonica* rice breeding program, and the demand for high quality rice is increasing along with increased interest in health and the nutritional quality of food (Kobayashi et al. 2008; Kwon et al. 2008; Lau et al. 2015). Therefore, many studies have been performed to understand eating quality or the relationship between eating quality and other traits while simultaneously attempting to improve these issues in rice. However, there are some difficulties in improving rice eating quality following the demand due to the evaluation methods, genetic complexity, interaction between genotypes and the environment, etc. The development and applications of DNA markers associated with eating quality are useful to address these problems and to increase the selection efficiency in the early breeding stage. Thus, in this study, we used a RIL population to assess whether TV, which means glossiness of cooked rice, improved eating quality. From QTL analysis, the QTL *qTV9* associated with eating quality was identified, and the dCAPS markers, including the target region delimited within the 34-kb segment, were developed. Three lines with *qTV9* were selected and confirmed that OE was maintained at the donor level in conjunction with TV, showing promising agronomic traits.

The RIL population derived from a cross between Hwayeong and Wandoaengmi6 was developed on the basis of the difference between the TV of parents and used for the genetic analysis on the glossiness of cooked rice. The phenotypic variation of the trait exhibited a normal distribution, associated by the QTL, and the mean value of RIL for the TV was significantly correlated each year (Table 1). In the correlation coefficiency for the 2 years, the tested traits involved physicochemical characteristics and starch viscosity indicated over 0.58 on average, while traits evaluated by the palatability test showed relatively low consistency (*r* < 0.27) across years.

In EFA performed for the tested 17 traits, the four factors were extracted to calculate the factor loadings with orthogonal rotation (Table 2). The OE along with TV and Pro in 2017 loaded on the first factor (PA1) and the OE (PA1) and the TV (PA2) were separated in 2018, but the correlation between both factors was still identified in 2018 (PA1:PA2, *r* = 0.3). In fact, the OE indicated a positive correlation with the TV for the 2 years (*r* = 0.48 in 2017 and *r* = 0.41 in 2018), and the Pro was negatively correlated with the OE for these years. Among the parameters in the palatability test, OE presented a relatively strong correlation with EQ, ST, and HD, and in the case of Amy, DH, and HR, the correlation was weak both years (Table 3). In general, many studies have reported a correlation between traits known to directly or indirectly affect rice eating quality. The texture parameters of cooked rice correlated with AC (Champagne et al. 2014). Sensory properties related to stickiness had statistical correlation coefficients with the AC and Pro (Lyon et al. 1999). Windham (2014) reported that it is very important to determine the correlation between the sensory and instrumental methods on palatability, which can lead better methods to quickly evaluate and predict end-use qualities. However, finding the relationships is not easy because the sensory properties of cooked rice are very subtle and can be influenced by many factors.. The result is meaningful in that the TV was more stable as an evaluation method for eating quality and showed less susceptibility to the growing environment in this study. Therefore, we think that TV obtained by an accurate instrument is appropriate for explaining the OE measured by the selected panel group.

In this study, a total of 14 QTLs associated with traits of eating quality were identified using QTL analysis (Table 4). Only the four QTLs, *qTV9, qPro9, qDH6*, and *qAmy3* on chromosomes 9, 6, and 3, were continuously detected for the experimental periods. Regarding the TV of cooked rice, three QTLs were detected on chromosomes 4, 6, and 9, indicating a positive influence on the alleles derived from Wandoaengmi6. In particular, the QTL *qTV9* delimited within Id9007180 and 9,851,330 on chromosome 9 was detected in both years, explaining approximately 17% of the variation on average in the ICIM analysis. The target region of *qTV9* was located within 20.83 to 21.28 Mbp, indicating the physical position (Fig. 4). The glossiness is known as an indirect parameter for eating quality based on the significant correlation between the palatability test and the glossiness of cooked rice (Takeuchi et al. 2007; Kwon et al. 2011). *qGCR6* affecting the glossiness of cooked rice from backcross inbred lines (BILs) developed from Koshihikari was identified on the short arm of chromosome 6 delimited to a 43.9-kb chromosomal region containing ten putative genes (Wang et al. 2017). Three QTLs derived from the *japonica* cultivar Ilpum in 2 years were identified on chromosomes 3 and 6, explaining a range of 7.5 to 28.0% (Cho et al. 2014). In particular, *qGCR9,* which is derived from the *O. rufipogon* allele, was associated with a higher glossiness of cooked rice and was reported within RM242 and RM245 on chromosome 9 (Yun et al. 2016). The physical position of *qTV9* detected in this study was included in the target region of *qGCR9* delimited within 18.64 Mbp and 22.04 Mbp on chromosome 9; in addition, although *qGCR9* is relatively weak, both QTLs were negatively related with the protein contents of cooked rice. On the other hand, the QTLs have something in common that the allele did not derived from normal *japonica* or *indica* cultivars.

To verify the effect of improvement in eating quality, the correlation coefficient between OE and TV was compared with the presence or absence of the detected *qTV9*. The positive effect on improvement of the OE was significantly confirmed in the introgression lines with *qTV9* (Fig. 3). From the results, the development of PCR-based markers was thought to be practically available to increase the selection efficiency by marker-assisted selection (MAS) for rice breeding programs on eating quality, even though *TV9* is the trait controlled by the QTL. To develop a DNA marker for MAS, we performed fine mapping of the target region of *qTV9*. To further narrow the region by the selected recombinants, *qTV9* was delimited to an approximately 34-Kbp segment flanked by the dCAPS markers CTV9_9 and CTV9_13, and 9 ORFs were listed in the target region as candidate genes associated with TV. Regarding ORFs listed in the target region, however, genetic information associated with grain formation or eating quality was not found in either previous relevant study or public database. In the further study we are planning to excess expression pattern of those at RNA level for confirming gene related to TV of cooked rice.

The CTV9_9 among developed markers was used to select the promising lines with the improved OE value, and then three lines within the introgression lines with *qTV9* were eventually selected (Table 5). In particular, the selected were maintained TV and OE without loss of favorable agricultural traits by a typical breeding method, such as resistance to BB and blast and panicle type (Table 5 and Additional file 8: Table S4). The values of the lines’ grain appearance also did not deviate significantly between the range of parents. In the case of physicochemical characteristics, the traits TV, OE, Pro and Amy of the lines tended to be somewhat close to Wandoaengmi6.

In this study, we tried to suggest that TV, which is obtained by the instrumental method, was one of the main traits explaining OE by statistical methods, and thus, we identified that *qTV9* was associated with the glossiness of cooked rice through QTL analysis. Moreover, *qTV9* was delimited within 34-Kbp on chromosome 9 by fine mapping, and a PCR-based DNA marker CTV9_9 associated with the QTL was developed using WGR data. In further study, the PCR-based DNA marker and selected lines will be practically applied for MAS breeding programs to improve eating quality in rice. Then, we will focus on characterizing the glossiness of cooked rice through functional analysis of the target region. In addition, we hopefully believe that this adds to the science surrounding rice eating quality and that molecular markers for the trait will prove valuable to breeding programs seeking to develop high quality rice lines with new sources.

## Materials and methods

### Plant materials and mapping population

Hwayeong, a mid-maturing *japonica* cultivar with high grain quality, and Wandoaengmi6, a *japonica*-type Korean weedy rice with high eating quality and a high degree of glossiness of cooked rice were crossed to develop a recombinant inbred lines (RILs) population using a single descent method (SSD). The population (F_8_) composed of 174 lines was used to access the phenotypic data of tested traits related to rice eating quality as well as to construct a molecular genetic map to identify the QTLs controlling eating quality.

### Evaluation of properties for eating quality

#### Physicochemical characteristics

The taste value (TV) is obtained by quantifying the glossiness of the surface of cooked rice. The value was measured from cooked rice left at room temperature for 5 min after cooking 33 g of head rice from each line at 80 °C for 10 min using a Toyo meter (MA-90, Toyo Rice Cleaning Machine Co., Ltd., Wakayama, Japan).

The amylose content was determined by the methods of Perez and Juliano (1978). Briefly, 100 mg rice flour left in a 95 °C dry oven for 5 h was gelatinized with 1 ml EtOH and 9 ml 1 N NaOH in a 97 °C water bath for 10 min. After cooling, distilled water was added up to 100 ml at room temperature. The 5 ml taken from the solution was mixed with 1 ml CH_3_COOH and 2 ml 2% lodine solution to a total of 100 ml and treated for 20 min. The value was measured from the solution at 620 nm using an Ultrospec 4300pro spectrophotometer (Amersham Bioscience, UK). The protein content was determined following the micro-Kjeldahl method (AOAC 2002). The ASV was determined visually using a score (1–7) of spreading according to the standard evaluation system for rice from the IRRI (IRRI 2013) and by the clearing of milled rice kernel soaked in 1.4% KOH solution for 23 h at a constant temperature of 30 °C.

#### Sensory test for evaluating eating quality

Using one cultivar—Shindongjin—as a standard, a check sensory test was carried out following the evaluation manual (Yamamoto et al. 1996). A total of 200 g of white rice polished at 92% degree of milling was prepared for the test. The white rice was washed 3–4 times and soaked for 30 min before cooking in an electric rice cooker (CUCKOO CR-0313 V, Seoul, Korea) at a ratio of white rice to water of 1:1.1 (w/v). The eating quality of cooked rice was evaluated to discriminate eating quality differences among tested lines by adult 7–15 panelists. The evaluation of eating quality was performed according to the six categories of research: AP: appearance and glossiness of cooked rice, AR: aroma of cooked rice, EQ: eating taste of cooked rice, ST: stickiness of cooked rice, HD: hardness of cooked rice, and OE: overall eating quality. The OE score was determined by summing the results of the five tests mentioned above. The eating quality of each tested item was scored from − 3 (extremely low) to + 3 (excellent) compared with that of the reference cultivar (score = 0). The mean score of all panelists was used as the trait value for the sensory test in this study.

#### Paste viscosity profile

The pasting properties of the rice flour were determined using a Rapid Visco Analyzer (RVA 4500, Perten Instruments, Stockholm, Sweden) according to the instruction method reported by the AACC method 61–02.01 (AACC International 1995). Three grams of rice flour at 14% moisture from each line was dispersed in 25 ml distilled water and subjected to gelatinization analysis. The sequential temperature curve was as follows: incubating at 50 °C for 1 min, heating at 95 °C for 1.4 min, cooling from 95 °C to 50 °C and then holding for 1.4 min. Four primary parameters, including pasting temperature (PT), peak viscosity (PV), hot paste viscosity (HV), and cool paste viscosity (CV), can be obtained from the pasting curve or temperature profile of the diagram. In addition, the secondary parameters of paste viscosity breakdown viscosity (BD) and setback viscosity (SB) were calculated; BD is the decrease in viscosity during cooking at 95 °C (BD = PV – HV), and SB is the viscosity (SB = CV – PV) when cooled to 40 °C min peak viscosity.

#### Evaluation of major agronomic traits

Head rice (HR) is the ratio of unbroken rice grain derived from a defined mass of paddy rice after complete milling. The HR was prepared following the method described by Tan et al. (1999) From the NICS standard evaluation system (RDA, 2012), major agronomic traits were evaluated; days to heading (DH) were calculated from the total number of days from seeding to 40% flowering, and culm length (CL) and panicle length (PL) were measured from 20 plants.

### Genotyping and linkage mapping

SNPs showing polymorphic patterns within the parents were surveyed using the 7 K Infinium SNP genotyping platform (Illumina®) at the Genotype Service Laboratory in IRRI (International Rice Research Institute, Philippines). The selected SNPs from the genotypic data sets decoded to generate SNP data using GenomeStudio Software were used to construct a linkage map using QTL IciMapping version 4.0 (Meng et al. 2015). The mapping distance during linkage map construction was calculated with the Kosambi mapping function, and the options By LOD and By Input were used for grouping and ordering of the selected factors, respectively.

### Data analysis

To detect the QTLs related to eating quality in rice, the phenotypic measurement and the genotypic SNP data were combined and analyzed using conventional mapping for inclusive composite interval mapping for additive QTLs (ICIM-ADD) (Zhang et al. 2008). Permutation tests with 1000 replicates (*P ≤* 0.05) were applied to confirm the significant threshold values (≥ 2.40 in 2017 and ≥ 2.55 in 2018) of the detected QTLs according to the method by Churchill and Doerge (1994). The naming of the QTLs followed the nomenclature suggested by McCouch 2008. The statistical packages, psych and ggplot2, in R software were applied to produce correlation coefficients and among tested traits, determination coefficients between TV and OE, and exploratory factor analysis (EFA). The corr.test () function produced correlations and significance levels for matrices of the Pearson correlations. In addition, the MASS package in R was used for *t*.test to compare to the mean value of traits within parents.

### Development of DNA markers using whole-genome resequencing

To harbor additional DNA markers within the target region, WGR was performed using an Illumina NovaSeq 6000 system (Illumina, USA) following provided protocols for 2 × 100 sequencing. The DNA library was prepared according to the Truseq Nano DNA library prep protocol (Cat. No. FC-121-4001). After QPCR using SYBR Green PCR Master Mix (applied Biosystems), the libraries that index tagged in equimolar amounts in the pool were combined. Using the resequencing data, the dCAP markers were designed by primer 3 by detecting the specific restriction site according to the sequence of parents in the target region.

## Supplementary information

**Additional file 1:** **Figure S1.** Comparison of the glossiness on the surface of cooked rice in the parents Hwayeong and Wandoaengmi6.

**Additional file 2:** **Figure S2.** Diagram of the oblique four-factor solution for the traits data associated with rice eating quality using the RILs in 2017 and 2018. Toyo is TV evaluated by a Toyo meter.

**Additional file 3:** **Figure S3.** Coefficients of pairwise correlations of 13 traits detected in eating quality of rice grain in the RILs in 2017. **Figure S4.** Coefficients of pairwise correlations of 13 traits detected in eating quality of rice grain in the RILs in 2018.

**Additional file 4:** **Figure S5.** Genetic linkage map of the RIL mapping population from a cross between Hwayeong and Wandoaengmi6, using 498 SNP markers.

**Additional file 5:** **Table S1.** Summary of the linkage map for QTL analysis.

**Additional file 6:** **Table S2.** List of dCAPs markers within the target region of *qTV9.*

**Additional file 7:** **Table S3.** List of putative ORFs in MSU and RAP version within the target region.

**Additional file 8:** **Table S4.** Grain appearance and other traits of selected lines and parents.

## Data Availability

Not applicable.
