# Supplementary figures and images for: Improving the Glossiness of Cooked Rice, an Important Component of Visual Rice Grain Quality
Source: Rice (N Y). 2019 Nov 27;12:87. doi: 10.1186/s12284-019-0348-0 (PMC6881499; doi:10.1186/s12284-019-0348-0)

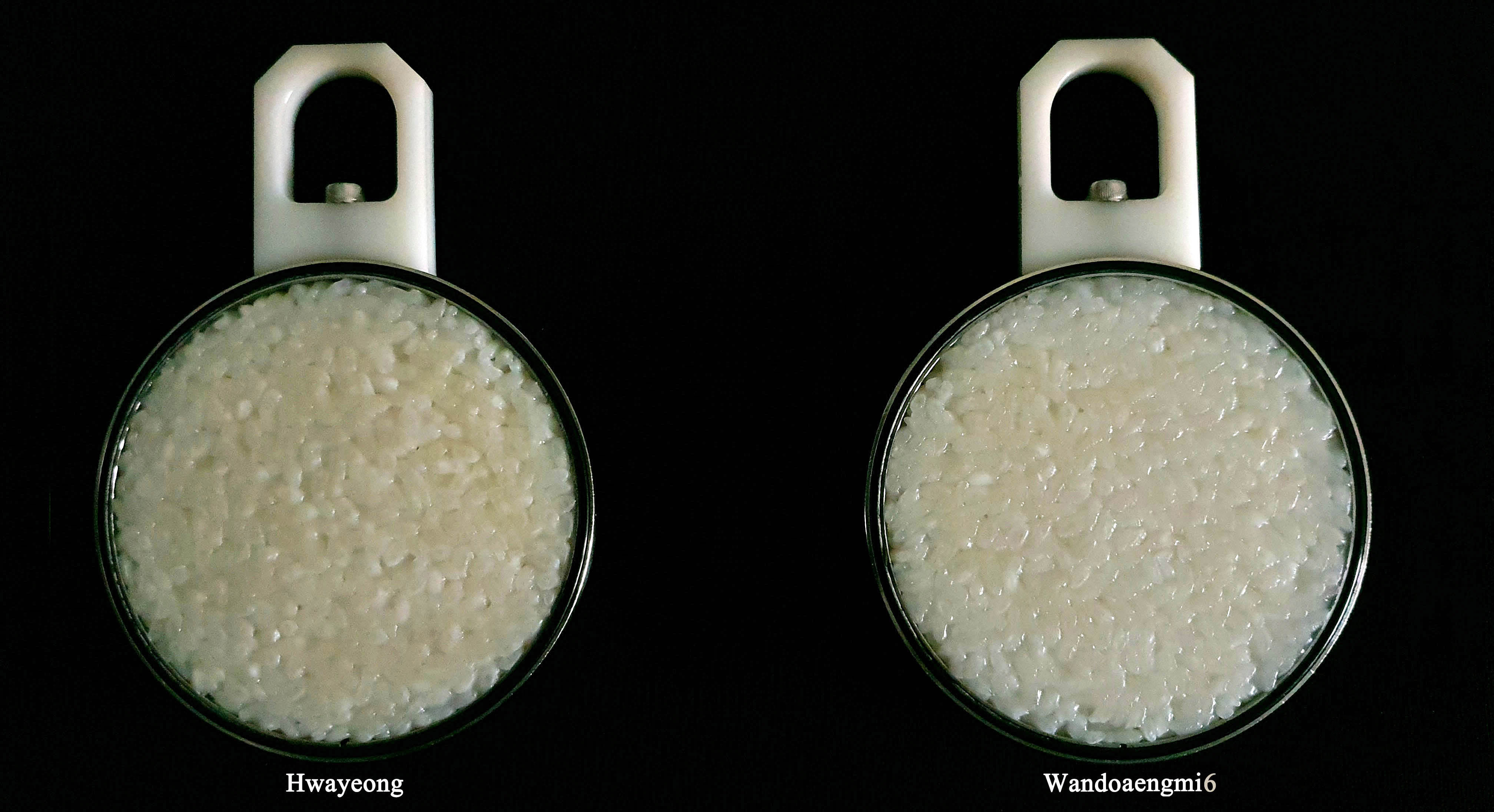

Supplement: Supplementary file 1 — Additional file 1: Figure S1. Comparison of the glossiness on the surface of cooked rice in the parents Hwayeong and Wandoaengmi6. [file 12284_2019_348_MOESM1_ESM.jpg]

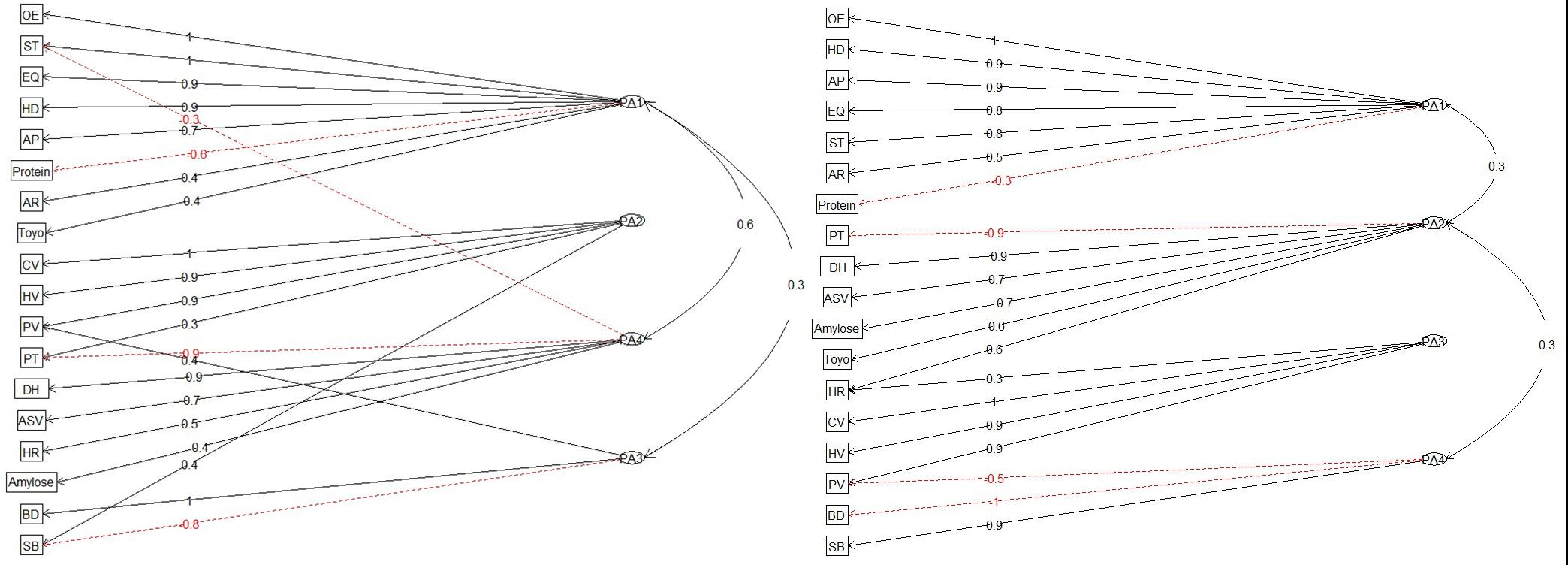

Supplement: Supplementary file 2 — Additional file 2: Figure S2. Diagram of the oblique four-factor solution for the traits data associated with rice eating quality using the RILs in 2017 and 2018. Toyo is TV evaluated by a Toyo meter. [file 12284_2019_348_MOESM2_ESM.jpg]

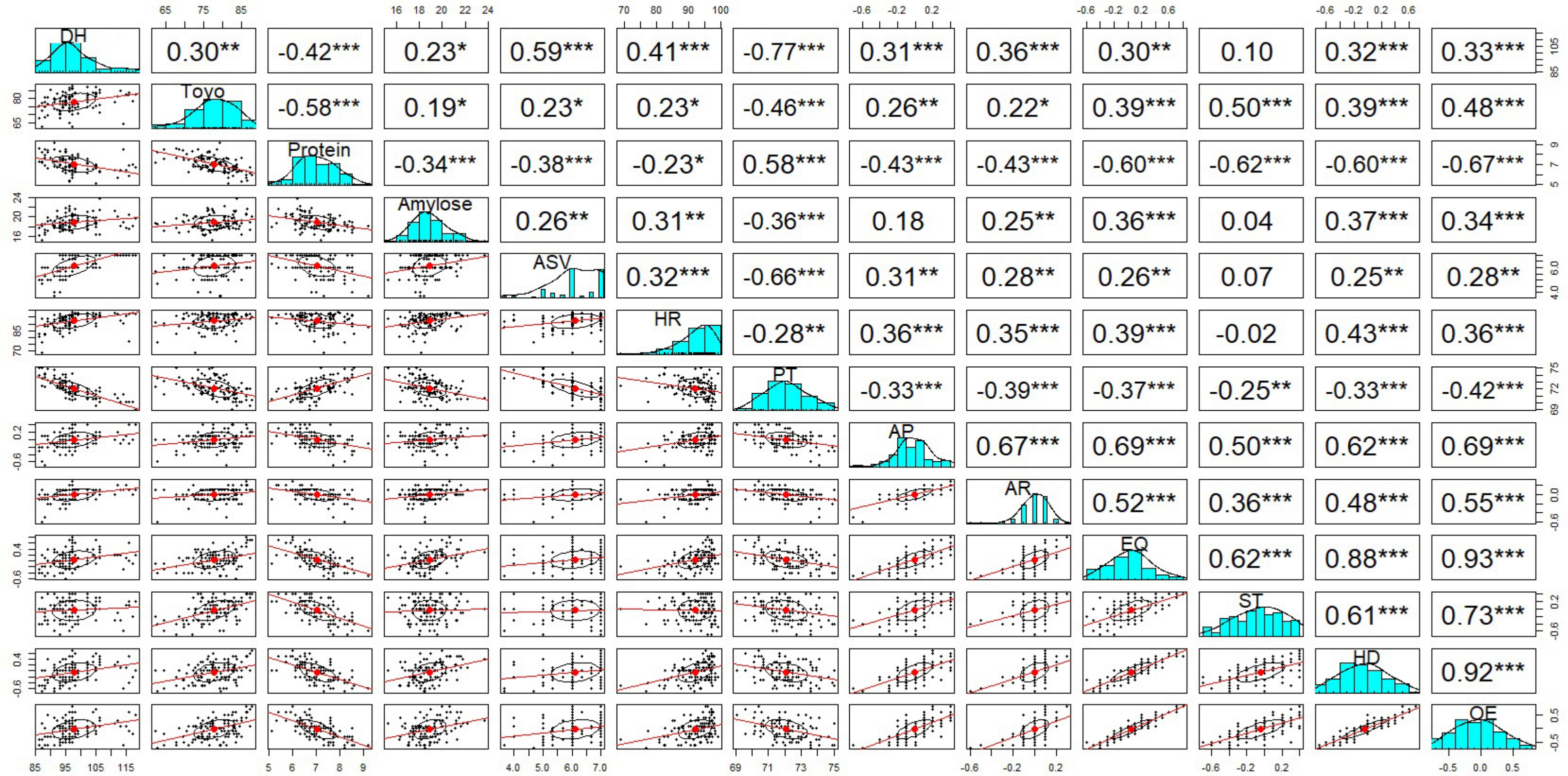

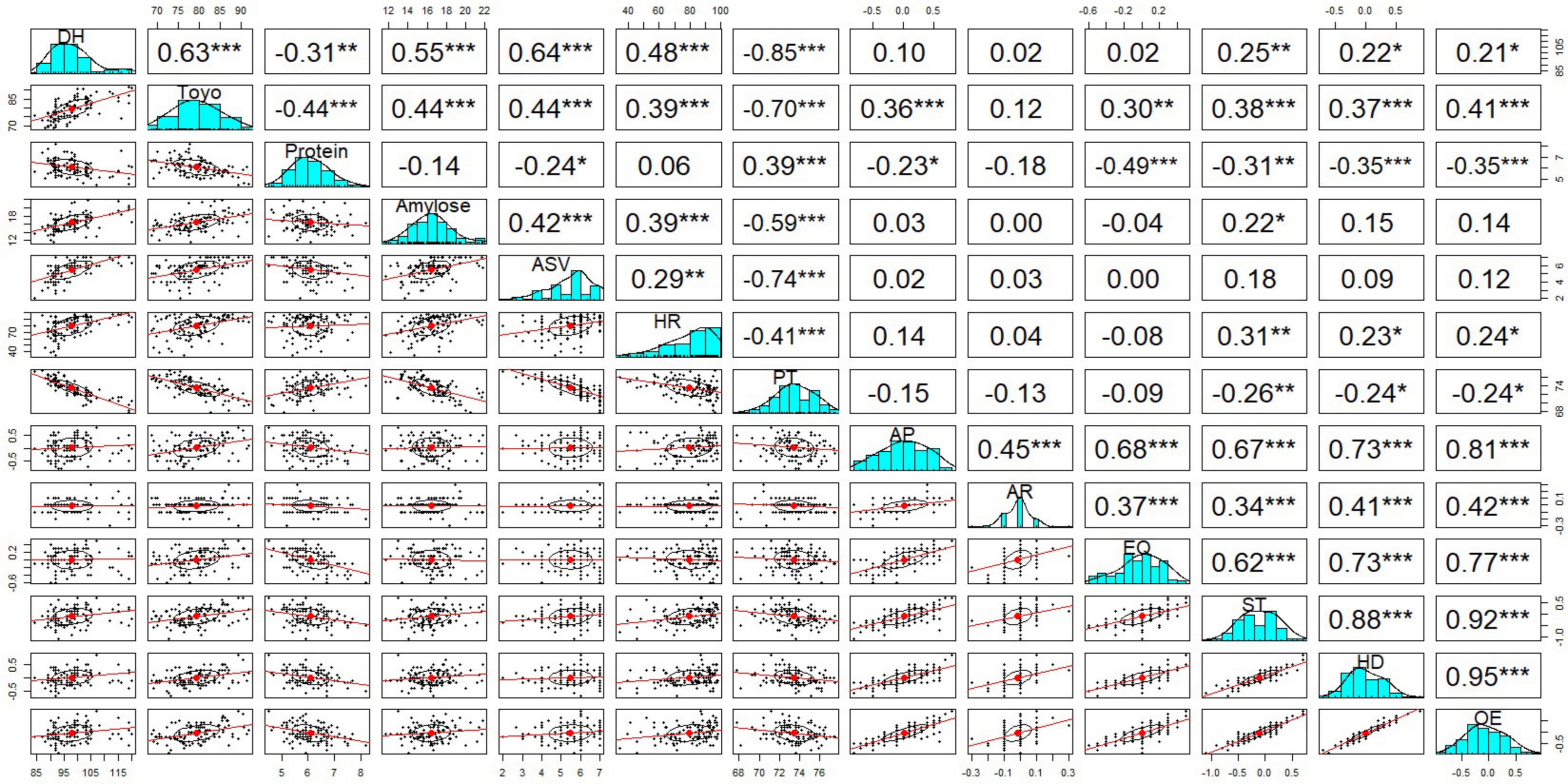

Supplement: Supplementary file 3 — Additional file 3: Figure S3. Coefficients of pairwise correlations of 13 traits detected in eating quality of rice grain in the RILs in 2017. Figure S4. Coefficients of pairwise correlations of 13 traits detected in eating quality of rice grain in the RILs in 2018. [file 12284_2019_348_MOESM3_ESM.pdf]

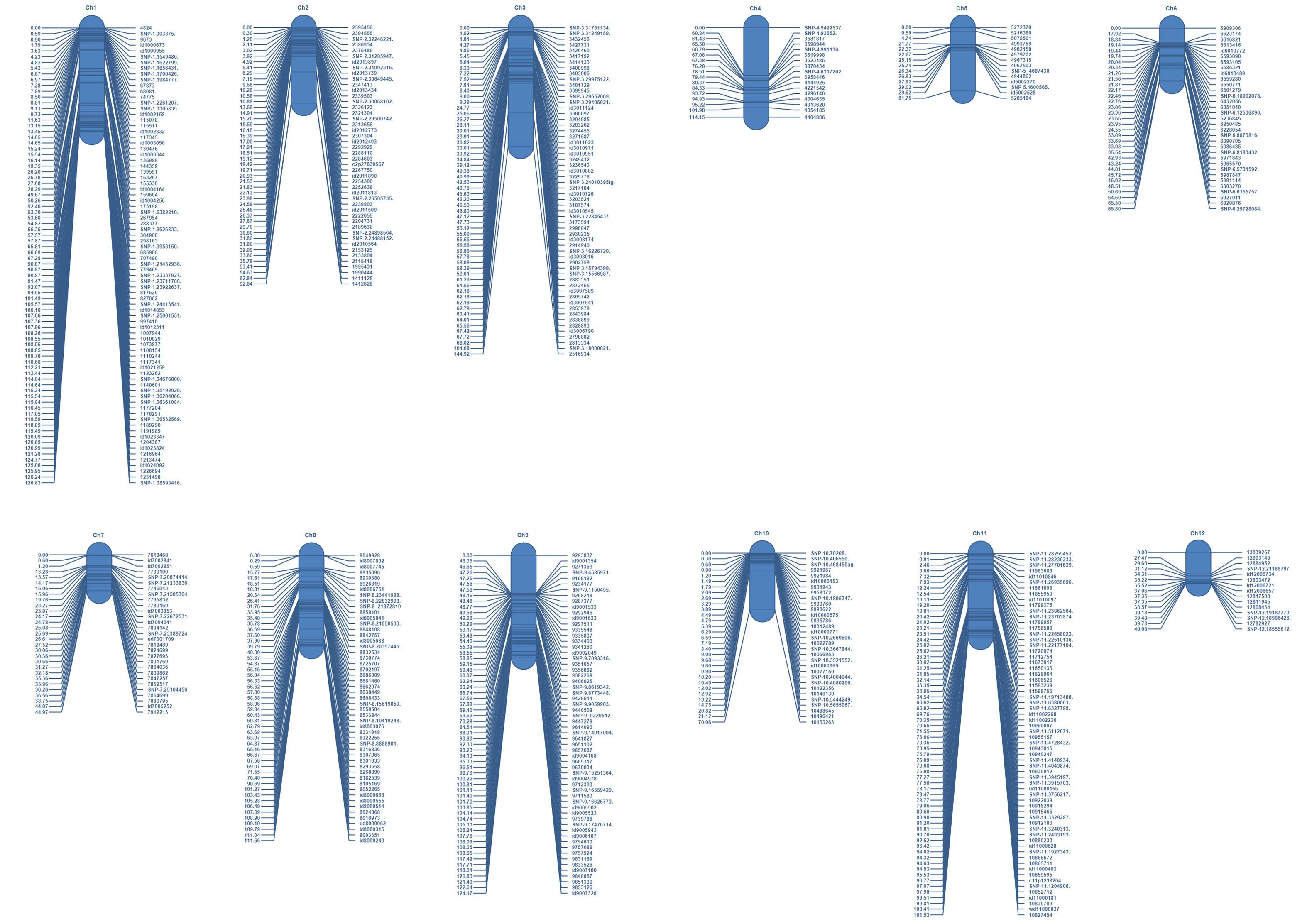

Supplement: Supplementary file 4 — Additional file 4: Figure S5. Genetic linkage map of the RIL mapping population from a cross between Hwayeong and Wandoaengmi6, using 498 SNP markers. [file 12284_2019_348_MOESM4_ESM.jpg]
