# Supplementary material for: Improving the Glossiness of Cooked Rice, an Important Component of Visual Rice Grain Quality
Source: Rice (N Y). 2019 Nov 27;12:87. doi: 10.1186/s12284-019-0348-0 (PMC6881499; doi:10.1186/s12284-019-0348-0)
Supplement: Supplementary file 5 — Additional file 5: Table S1. Summary of the linkage map for QTL analysis. [file 12284_2019_348_MOESM5_ESM.pdf]

Supplementary Table S1. Summary of the linkage map for QTL analysis

Chromosome	Used markers	Polymorphic markers	Polymorphism (%)	Anchored markers [a)]	Length [b)] (cM)	AMI [c)] (cM)
1	850	150	17.66	80	125.11	1.6
2	660	88	13.33	46	94.12	2.0
3	683	102	14.93	57	116.93	2.1
4	646	28	4.33	18	55.76	3.1
5	509	27	5.30	14	108.32	7.7
6	551	106	19.23	33	77.22	2.3
7	524	60	11.45	29	45.31	1.6
8	545	98	17.98	51	110.63	2.2
9	455	133	29.23	60	125.08	2.1
10	472	73	15.46	31	66.68	2.2
11	631	128	20.38	64	99.72	1.6
12	572	31	5.41	15	39.94	2.7
Total (average)	7098	1024	14.42	498 (41.5)	1064.82	2.1

[a)] Number of markers selected
[b)] Length: chromosomal length
[c)] Average marker interval
