# Supplementary material for: Improving the Glossiness of Cooked Rice, an Important Component of Visual Rice Grain Quality
Source: Rice (N Y). 2019 Nov 27;12:87. doi: 10.1186/s12284-019-0348-0 (PMC6881499; doi:10.1186/s12284-019-0348-0)
Supplement: Supplementary file 6 — Additional file 6: Table S2. List of dCAPs markers within the target region of qTV9. [file 12284_2019_348_MOESM6_ESM.pdf]

Supplementrary Table S2. List of CAPs marker within the target region of *qTV9*

Marker name	Polymorphism*	Position (bp)**	TM. (°C)	Restrict enzyme	Incubation TM.	Target site	F	R
CTV9_1	X	21160068	58	Hpy188I	37	TCNGA	GAGTCTAGTGTACACCGCTGG	TCGTTTCGCTTAGTTCAG
CTV9_2	O	21192039	50	EcoRV	37	GATATC	TTTAATCCTTAATAGGGATAT	GCCCATCTGGAATTTGTCATGG
CTV9_3	O	21196113	50	SpeI	37	ACTAGT	TGCATCAAATGTGGAGCGAC	GAAATCAAGTCTGACTA
CTV9_4	X	21197336	58	EcoRII	37	CCWGG	CCCCCGACTATAGCTCGAACC	GTGGAAGGAGTCGGAGTTGG
CTV9_5	X	21205423	50	ApaLI	37	GTGCAC	TGACTCAACAAGTG	AACGCGTGAAGGATGTTTGC
CTV9_6	O	21206559	50	RsaI	37	GTAC	TCTGCAGAATCTTAGTA	ACTTTCGTTGATGGACCGAA
CTV9_7	X	21211307	58	SpeI	37	ACTAGT	GGAGTTTGGAAGGAGCAATGCACT	AGCCAGAGACCCCCTATGAG
CTV9_8	X	21223863	58	MseI	37	TTAA	CAGAAGAGGTGAGAAGGCCG	TGTATAAAAGTTTTGATAAGATT
CTV9_9	O	21248273	58	Rsal	37	GTAC	GGGGAAGGGTCTAGAAGGGT	TGTTCTCTTATGTTAAAAGGTCGG
CTV9_10	X	21255230	50	HinfI	37	GANTC	ATCTTTTAAGATTGTGAGTAGAA	GTGGCTTGGACTGATATCTGGT
CTV9_11	X	21273493	58	Hpy188I	37	TCNGA	AATGGAAGGTTGTCGG	GGCCAGCAGTAAGCCAAGTA
CTV9_12	X	21281014	50	MseI	37	TTAA	AAGGTTGAAAATTTAAATTTA	GTCTCGCTCTCTGGCTTGTT
CTV9_13	O	21282422	58	HinfI	37	GANTC	TGGAACGATGTGACGGAAGAGT	CACCGGTCCATTTTCATTTCGA
CTV9_14	X	21285122	58	SpeI	37	ACTAGT	AATTAAGACCTCCAGTAAC	CTCCCAGTCCCAGCAAAGAG
CTV9_15	X	21286885	58,50	MseI	37	CTAG	TGTGTAAGAAAGTTTA	AGCCTGGCGAGTTAGGATTT
CTV9_16	X	21287524	58,50	MseI	37	TTAA	TACAAACTTTCAATTTTTA	GGAAGGAGTATCATACTAGTACGGG

* indicate the presence of qTV9 (O: positive, X: negative qTV9)

** is physical position on chromosome 9
