# Supplementary material for: Improving the Glossiness of Cooked Rice, an Important Component of Visual Rice Grain Quality
Source: Rice (N Y). 2019 Nov 27;12:87. doi: 10.1186/s12284-019-0348-0 (PMC6881499; doi:10.1186/s12284-019-0348-0)
Supplement: Supplementary file 7 — Additional file 7: Table S3. List of putative ORFs in MSU and RAP version within the target region. [file 12284_2019_348_MOESM7_ESM.pdf]

Supplementary Table S3. List of putative ORFs in MSU and RAP version within the target resion

#	Locus (MSU)	Futative fuction	#	Locus (RAP)	Futative fuction
1	LOC_Os09g36840	Expressed protein	-	-	-
2	LOC_Os09g36850	Expressed protein	1	Os09g0539700	Conserved hypothetical protein
3	LOC_Os09g36860	acyl carrier protein, putative, expressed	2	Os09g0539800	Similar to Acyl carrier protein III, chloroplast precursor (ACP III)
-	-	-	3	Os09g0539901	Hypothetical gene.
-	-	-	4	Os09g0540000	Hypothetical gene
-	-	-	5	Os09g0540150	Conserved hypothetical protein
4	LOC_Os09g36870	plant protein of unknown function domain containing protein, expressed	6	Os09g0540300	Protein of unknown function DUF247, plant domain containing protein
-	-	-	7	Os09g0540325	Hypothetical protein
-	-	-	8	Os09g0540350	Non-protein coding transcript
5	LOC_Os09g36880.1	GDSL-like lipase/acylhydrolase, putative, expressed	9	Os09g0540400-1	Similar to Anther-specific proline-rich protein APG
6	LOC_Os09g36880.2	GDSL-like lipase/acylhydrolase, putative, expressed	10	Os09g0540400-2	Similar to Family II lipase EXL3.
7	LOC_Os09g36890.1	hydroxyproline-rich glycoprotein family protein, putative, expressed	11	Os09g0540500	Mediator complex, subunit Med4 domain containing protein
8	LOC_Os09g36890.2	hydroxyproline-rich glycoprotein family protein, putative, expressed	-	-	-
9	LOC_Os09g36900	WD domain, G-beta repeat domain containing protein, expressed	12	Os09g0540600	Similar to WD-40 repeat protein MSI1

Transcription evidence
-
AK242905
AK058903
CU861776
AK064703
AK242511
ab initio prediction
EU956917
AK288529
AK111927
AK112006
AK120729
-
AK103397:AK101798
