# Supplementary material for: Improving the Glossiness of Cooked Rice, an Important Component of Visual Rice Grain Quality
Source: Rice (N Y). 2019 Nov 27;12:87. doi: 10.1186/s12284-019-0348-0 (PMC6881499; doi:10.1186/s12284-019-0348-0)
Supplement: Supplementary file 8 — Additional file 8: Table S4. Grain appearance and other traits of selected lines and parents. [file 12284_2019_348_MOESM8_ESM.pdf]

Supplementary Table S4. Grain appearance and other traits of selected lines and parents

Parents & Lines	Length (mm)	Width (mm)	L/W	Thickness (mm)	BL	BB (K1)	Panicle type
Hwayeong	7.17c	3.15a	2.27c	2.29a	M	R	Normal
Wandoengmi6	7.78a	2.96b	2.61b	2.04b	R	R	Compact
HW148	7.77a	3.03b	2.57b	2.09b	R	R	Normal
HW184	7.63a	2.75c	2.77a	1.93c	R	R	Normal
HW191	7.50b	2.82c	2.66ab	1.88c	R	R	Normal
